# Management of Nasal Septum Chondrosarcoma Occurring in Elderly: A Case Report

**DOI:** 10.7759/cureus.1497

**Published:** 2017-07-20

**Authors:** Liliana Belgioia, Elena M Vaccara, Almalina Bacigalupo, Renzo Corvò

**Affiliations:** 1 Radiation Oncology, University of Genoa Ospedale Policlinico San Martino; 2 Medical Physics, University of Genoa Ospedale Policlinico San Martino; 3 Radiation Oncology, University of Genoa Ospedale Policlinico San Martino

**Keywords:** nasal septum chondrosarcoma, helical tomotherapy, imrt, elderly

## Abstract

Chondrosarcomas (CS) are tumors affecting mainly pelvis, long bones and ribs in middle aged people; involvement of head and neck is rare and nasal septum location is exceptional. We report the follow-up of an 85-year-old patient suffering from head and neck CS. Magnetic resonance imaging (MRI) showed a mass originating from nasal septum. The patient underwent to debulking surgery and adjuvant hypofractionated radiotherapy with excellent tolerability and no evidence of recurrence after 16 months. Hypofractionated radiotherapy delivered with advanced technique (helical tomotherapy) allowed to deliver high doses radiation in elderly patients with limited toxicity.

## Introduction

Chondrosarcomas (CS) are non-epithelial malignant, slow growing tumors that usually involve pelvis, ribs, and long bones of extremities [1]. They mostly occur in middle-aged people and etiopathogenesis remains unknown. CS hit 10-20% to primary bone, some 5–10% to head and neck region and rarely the nasal septum [1]. Treatment is primarily surgical; adjuvant therapy is recommended if a radical resection is not possible [2]. This paper presents an 85-year-old man's case with CS nasal septum that has been treated with endoscopic debulking surgery and adjuvant photon radiotherapy with excellent tolerance and outcomes.

## Case presentation

**Figure 1:**
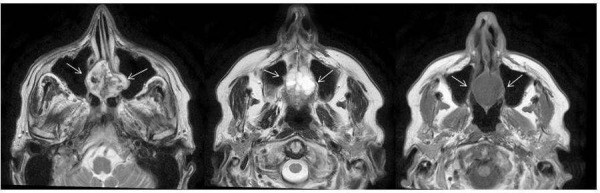
Magnetic resonance imaging (MRI) scan at diagnosis. The arrowheads indicate the lesion.

**Figure 2:**
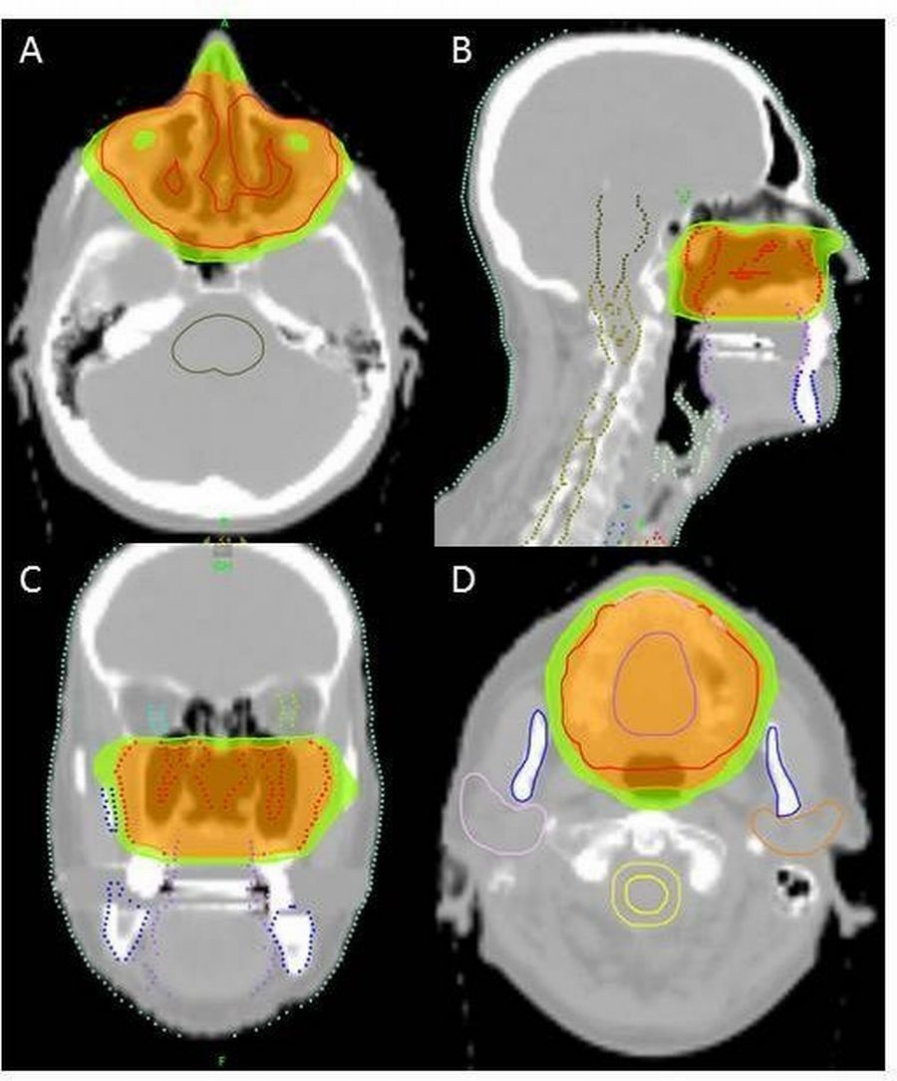
Intensity modulated radiotherapy (IMRT) treatment planning by helical tomotherapy. In orange 95% isodose, in green 80% isodose. In figure (A) and (D) axial images, in (B) sagittal image and in (C) coronal image.

**Figure 3:**
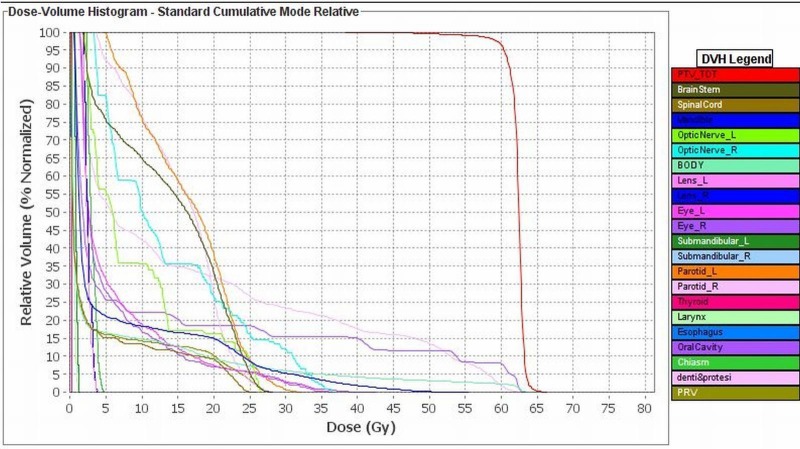
Dose-volume histogram of the dose to the target volume and organs at risk

The patient reported history of nasal obstruction, mainly at right side since August 2015. Nasal endoscopic examination revealed a large submucosal mass of nasal septum with occlusion of the posterior third and extending to hard palate on the left side. Computed tomography (CT) scan detected a mass of about 4 cm with extension across the midline and erosion of the nasal septum, laterally extending into maxillary sinus with infiltration of the left one and invading inferiorly the hard palate especially on the left side. Magnetic resonance imaging (MRI) confirmed these data (Figure 1) and the subsequent biopsy revealed a CS. Systemic staging did not show any evidence of loco-regional or distant metastases. On October 2015, the lesion was resected endoscopically under general anesthesia, despite patient's old age as in his past medical history no important comorbidity was referred with the exception of atrial fibrillation, arteriosus hypertension and a recent pneumonia completely resolved. The intent of surgery was mainly to debulk major CS with expected residual disease. Definitive histopathological examination confirmed the preoperative diagnosis of grade II CS with the evidence of positive margins on hard palate and sphenoid rostrum. Due to the old age and logistic aspects, the patient refused to be seen at proton therapy center to be evaluated for treatment with particle radiations. On January 2016, the patient underwent to adjuvant radiation treatment, with helical intensity modulated radiotherapy (IMRT) technique and daily image guided radiotherapy (IGRT) by Tomotherapy. A CT simulation scan was acquired with slices of 2.5 mm from the vertex to clavicular bone and clinical target volume (CTV), its 5 mm expansion – planning target volume (PTV) – and all organs at risk (OARs) such as brainstem, spinal cord and its 5 mm expansion planning risk volume (PRV), temporomandibular joint, mandible, optic nerve, lens and eyes, parotids, larynx, esophagus, oral cavity, chiasm, prosthesis were contoured by radio-oncologist expert in ear, nose and throat (ENT) cancers. Tomotherapy needs more volumes definition in respect to 3-dimensional conformal radiotherapy (3DCRT): in addition to the organ at risk, “help structure” is necessary to optimize the dose distribution. The prescription dose to PTV was 62.5 Gy in 25 fractions, 2.5 Gy per fraction. By assuming the α/β radio sensitivity value of 2.5 for CS, the estimated biological tumor dose (133 Gy) elapsed with this fractionation schedule was equivalent to 74 Gy in 2 Gy/fraction. The radiotherapy plan was calculated with fine grid, the smallest field available width of 1.05 cm, a high modulation factor of 3.7 and a pitch of 0.287. The high modulation and the smallest field together with an increase of the treatment beam-on time that is 11.7 minutes provided an excellent conformity to the target of the isodose curves (Figure 2). The strategy was to divide the PTV into two regions: the upper PTV close to the eyes (PTV1) and the lower PTV overlapping with the oral cavity (PTV2). Our priority was to have optimal homogeneity and no hot spots on PTV2, while the coverage of PTV1 was slightly sacrificed in favor of sparing the eyes (Figure 3). The treatment was well tolerated with grade 1 mucosal and skin acute toxicity according to Radiation Therapy Oncology Group (RTOG) scale. After two weeks, the mild pain was completely resolved. No late toxicity recorded. At the last follow-up, 16 months after the radiotherapy ended, there is no evidence of recurrence.

## Discussion

CS to the head and neck region are rare, with few series from a single institution being reported. The most common sites in this district are the larynx, maxilla and skull base; lesions arising from the nasal septum are, therefore, extremely rare [1]. Generally, head and neck CS is a disease with a median age at diagnosis in the fourth decade or about 10 to 20 years earlier than for all other body position [1]; in contrast to this datum, our patient is very old for this type of disease. Review of English-written literature from 1927 to date lists 63 CS nasal septum cases and, of these, only four were in aged patients (>70 years, two of four over 80 years). The clinical presentation occurs when surrounding structures as orbits, paranasal sinus or cranial fossa are involved; usually, as a first sign, patients present with persistent nasal obstruction, as in our case. From a histological point of view, CS may be classified into three grades on the basis of the degree of cellularity, nuclear size, atypia and mitotic activity [3]. Grade 1 CS present abundant chondroid matrix, clusters of chondrocytes with normal or slightly irregular nuclei, rare nucleoli, absence of mitoses, and occasional binucleation. Grade 2 CS exhibit less chondroid matrix and more chondrocytes than grade I tumors, presence of rare mitoses, slightly enlarged vesicular hyperchromatic nuclei, and multinucleation. Finally, grade 3 tumors display high cellularity, a myxoid matrix with irregularly shaped chondrocytes, prominent nuclear pleomorphism, and increased mitoses compared with the low-grade CS [3]. Grade is a key prognostic indicator according to the American Joint Committee on Cancer Staging (AJCC); the five-year survival rates for all histologic grades ranged from 54% to 81% [1,4]. The real extension of disease and plan for the correct therapy are crucial to understand diagnosis imaging. Moreover, CS demonstrate characteristic findings on both CT and MRI. That differentiates CS from other lesions such as chondroma, meningioma or osteoblastoma, even if histological assessment remains mandatory [1]. Surgery is the primary mode of treatment: the goal of surgery is to obtain histology clear margins, as the reported relapse rate with “positive margins” rises up to 65% [5]. In our patient, the definitive histologic exam revealed R1 margin and, in a multidisciplinary discussion, a second resection has been excluded due to the patient age and, especially, to obtain a definitive radicalization a wide and aggressive destructive surgery became necessary so the patient was submitted to adjuvant RT. According to literature, proton therapy could have been a primary option, but the patient refused to move far away from his residency. CS are still considered fairly radioresistant tumors and, according to literature, CS require doses greater than 60 Gy to achieve local control after incomplete resection [6]. Even if hypofractionated, RT is not the standard in HN district, we chose a hypofractionated schedule to obtain a higher EQD2 taking into account the biological resistance of CS; moreover, a slight hypofractionation was reported also by other authors in the literature [3]. However, considering the diseased spot, RT may be linked to considerable morbidity if delivered with conformal technique. Use of helical tomotherapy allowed our treatment to obtain an excellent dose distribution (the 95% isodose line covers the 98% of PTV) and the plan was satisfactory due to the very high conformation of helical IMRT technique, which allows to spare critical OAR in proximity of PTV and to control the dose received by OAR within the PTV, such as oral cavity. The modified Homogeneity Index (mHI = (D5-D95)/PD, where D5 and D95 represent the doses received by the 5% and 95% volumes of PTV, respectively as suggested in [7]) was kept as low as possible for both PTVs compatibly with an optimal coverage; in particular, the resulting mHI value was respectively 0.05 for PTV1 and 0.04 for the smallest PTV2. Check of daily shifts was assessed using Mega Voltage CT (MVCT), indeed, the auto-matching software suggests four shifts from current and planned position that can be corrected on-set. During the treatment, we obtained the following results in terms of mean and standard deviation compared to the first fraction as LAT = -0.4 ± 2.5 mm, LONG = -1.4 ± 2.0 mm, VERT = 0.2 ± 1.2 mm and ROLL = 1.1° ± 1.1°.

## Conclusions

The peculiarity of our case study was the patient age, the rarity of tumor location and the RT schedule. Some literature data reported unfavorable outcomes in older patients. A definitive conclusion cannot be reached with so few cases; we had good short term outcomes with combined treatment, excellent feasibility, and limited toxicity. We recognize the necessity of lifelong follow-up as late recurrence is likely, due to the prolonged natural history of the disease. As an alternative option to particle radiotherapy, slightly hypofractionated photon delivery with advanced IMRT may be of benefit in elderly patients with acceptable toxicity. Adaptive radiotherapy by small and stable daily IGRT shifts may further increase the treatment reliability and could maximize the probability of a long-term clinical advantage.
